# From structural recognition to mechanistic forecasting: repositioning artificial intelligence in osteoarthritis prevention

**DOI:** 10.3389/fimmu.2026.1809658

**Published:** 2026-04-13

**Authors:** Qinghan Li, Yifan Wang, Minglei Zhang

**Affiliations:** 1 Department of Xinmin Orthopedic, China-Japan Union Hospital of Jilin University, Changchun, China; 2 Senior Department of Orthopedics, The Fourth Medical Center of Chinese People’s Liberation Army (PLA) General Hospital, Beijing, China

**Keywords:** artificial intelligence, chronic inflammation, early prevention, immunometabolism, osteoarthritis

## Introduction

Osteoarthritis (OA) is still the main cause of chronic pain and disability in the world. With the aging of population and the increase of metabolic disorder, it is predicted that the number of knee OA cases will increase by about 75% by 2050 (1). Although diagnostic imaging and surgical techniques have made great progress in the past decade, the overall disease burden is still increasing.

Artificial intelligence (AI) has made great contributions to structural evaluation. At present, the performance of deep learning system is close to the expert level when Kellgren-Lawrence (K-L) classification and cartilage segmentation are automatically performed (2–4). In surgery, AI-aided planning and robot system improve implant positioning and surgical accuracy (5). These advances represent important technological development.

However, most AI applications are mainly used to find and measure the structural damage that has occurred. They make the measurement more accurate, but they don’t really help us to start prevention earlier. Although the accuracy has improved, there are still many people suffering from OA and people who are disabled as a result. From this point of view, technological progress has not allowed us to stop the disease earlier. The limitation may not lie in algorithmic performance, but in where along the disease trajectory artificial intelligence is being applied. Reconsidering this alignment may be essential if AI is to meaningfully contribute to prevention rather than merely improve detection. From this point of view, we define mechanistic forecasting as a model framework, which combines long-term exposure history, multimodal biological signals and time dynamic changes of diseases to predict everyone’s risk trajectory. Unlike enhanced structural prediction, which mainly predicts or detects the severity of imaging, the goal of mechanistic forecasting is to simulate the upstream biological imbalance and its changes over time. Its purpose is not only to stage the disease, but to find the key turning point in the development of the disease before irreversible structural damage occurs.

## Artificial intelligence models remain anchored to structural phenotypes

Radiographic severity frequently shows weak and inconsistent associations with pain and functional limitation (6, 7). Some people’s joints have been severely deformed, but the symptoms are very mild; Others have been in severe pain before seeing obvious imaging changes.

This inconsistency suggests that structural phenotypes capture only part of the disease process. Pain perception in OA may be influenced by heterogeneous mechanisms, including inflammatory signaling, central sensitization, and interindividual variability in nociceptive processing. Therefore, structural alterations may not fully represent the temporal onset of underlying biological dysregulation.

More and more studies have found that the pathogenesis of OA may be related to mild chronic inflammation, metabolic disorder, innate immune activation and cell aging (8, 9). Synovitis and macrophage activity may appear earlier than obvious cartilage damage. Aging-related secretory phenotype (SASP) can also make inflammatory signals in joint tissues persist.

Recent machine learning analyses combined with lipid metabolism and inflammatory mediators found that we can divide the severity of diseases into different levels through the characteristics of metabolism-immunity (10). There are also some methods called high-dimensional clustering, which also find completely different types of diseases in biology among patients who seem to have similar diseases (11).

Generally speaking, these findings tell us that the structural damage we can see is often the result of later biological processes inside the body. Therefore, those AI systems that are trained only by image data actually learn what a disease looks like in the late stage, rather than how the disease developed at the beginning.

## Artificial intelligence rarely integrates exposure and temporal biology

The immune disorder in OA develops slowly. Obesity-related adipokines can affect the inflammation of joint synovium, abnormal mechanical load can change the expression of inflammatory genes through force conduction pathway, and the change of intestinal flora may affect the inflammatory state of the whole body (12). Population studies have shown that lifestyle and environmental exposure have a great influence on the risk of arthritis (13, 14).

These effects will accumulate over time, and their effects will influence each other and change constantly, thus shaping the immune response and tissue repair ability, but these are difficult to see through a single examination.

However, most AI applications in OA still rely on single image data (15, 16). Long-term environmental exposure history and immune characteristics are rarely integrated into a unified analytical framework. Although the biological model of OA emphasizes the cumulative and dynamic process more and more, the calculation model is often static. This mismatch may limit the role of AI in early prevention.

## Artificial intelligence should evolve toward risk trajectory modeling

**Figure 1:**
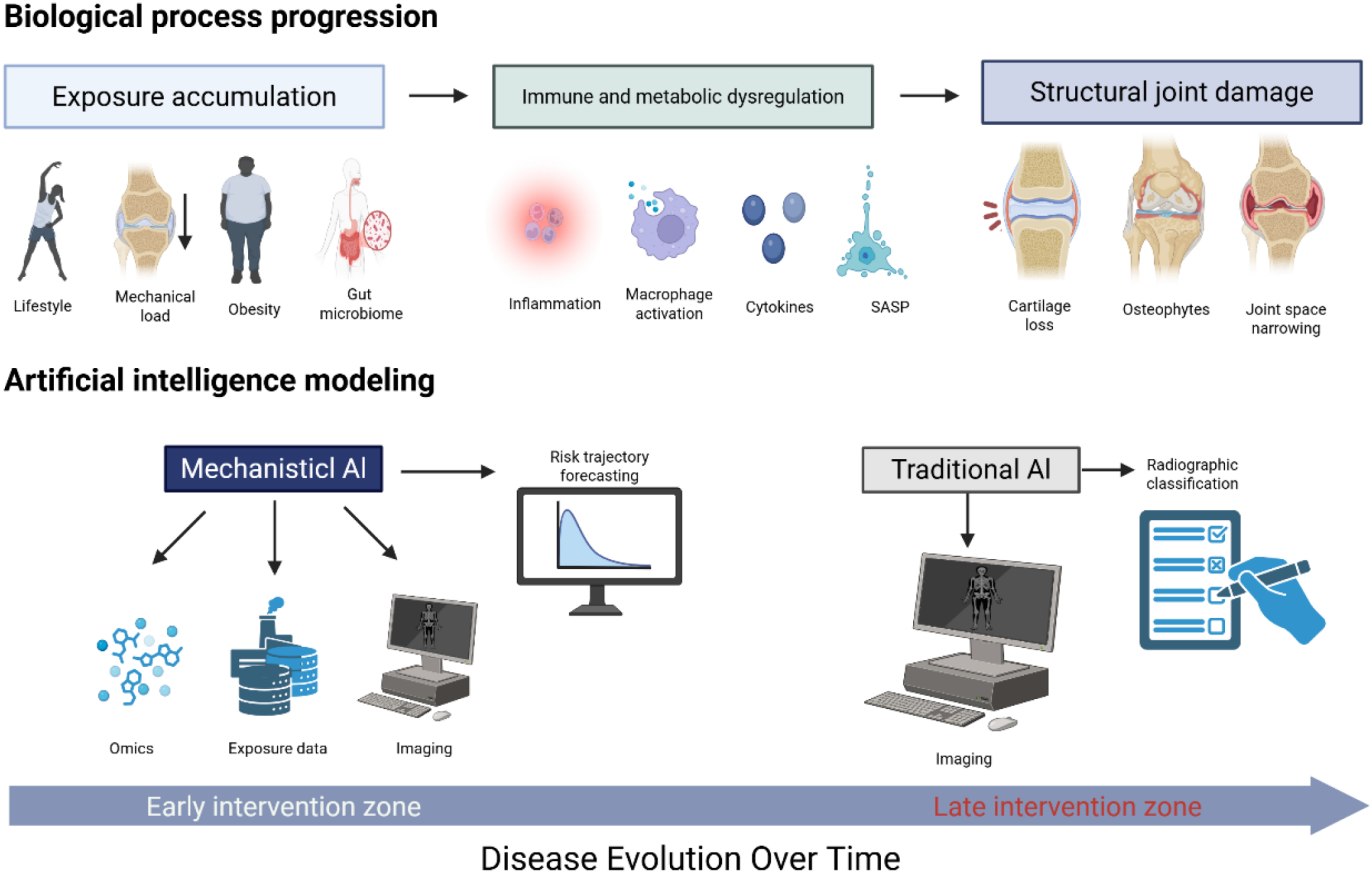
From structural recognition to mechanistic forecasting in osteoarthritis.

The proposed mechanistic forecasting framework for OA represents a paradigm shift from existing AI applications in this field. Unlike conventional AI models that solely rely on cross-sectional radiographic data for late-stage structural recognition, our framework innovatively integrates multimodal longitudinal data—including immune signatures, metabolic profiles, and long-term environmental exposure history—to model dynamic disease risk trajectories. Another key novelty is its shift from static disease classification to causal reasoning and intervention prediction, which links biological imbalances to actionable preventive strategies rather than merely identifying structural damage. This framework thus redefines AI’s role in OA from a passive detection tool to an active platform for anticipatory prevention. Notably, repositioning AI in the OA field does not require abandoning imaging data; instead, it demands an expansion of modeling objectives and input data dimensions. To elaborate on how AI can be repositioned along the temporal trajectory of osteoarthritis development, we propose a conceptual framework that directly maps the biological progression of the disease to corresponding modeling strategies. (**Figure 1**).

The multimodal method combining imaging, molecular characteristics and long-term exposure data is technically feasible. In practical implementation, the multimodal data integration follows a biology-driven attention-based architecture: radiographic structural features, immune-metabolic molecular signatures and long-term exposure data are first standardized and mapped to spatiotemporal disease nodes. The model then assigns dynamic attention weights to each data modality based on its biological relevance to OA progression and fuses these weighted features to construct individualized risk trajectories. This integration strategy ensures that non-imaging biological and environmental factors, which drive early OA pathogenesis, are incorporated into the core decision-making process of the AI model. Attention-based multimodal architectures have demonstrated the ability to dynamically model cross-modal relationships, suggesting technical feasibility for integrating imaging, molecular, and exposure data in OA. However, the biological relevance of input variables may be more important than simple architectural complexity.

It is also important to shift from static classification to time prediction. Traditional tasks (such as K-L grading) provide cross-section labels. The longitudinal modeling method can estimate the individual’s progress pattern with time (17). The model no longer merely stratifies disease stages but can generate an individualized risk curve and predict the probability of future pain aggravation or structural deterioration.

Unsupervised clustering technology may also help us find some immune subtypes caused by environmental factors (11, 18). This classification can reduce the complexity of the situation and help us to formulate more targeted preventive measures.

However, the accuracy of prediction is not enough. The prediction model is mainly used to calculate the statistical relationship between input variables and results. But even if it is predicted accurately, it does not mean that changing a variable can affect the development of the disease. On the contrary, the causal reasoning framework will try to find out the factors that can really change the disease, rather than those indicators that are only related. The intervention model goes further. It can estimate how the future risks will change after changing some exposure factors or biological states. If these distinctions are not made, even if the AI system predicts accurately, it may be of limited help to prevention decision-making.

## Translational and methodological constraints remain substantial

Mechanical prediction brings great complexity. We need a long-term tracking queue infrastructure to collect standard exposure measurement data. Multiomics integration requires strict data coordination and careful handling of missing data. In clinical application, the model must be interpretable, calibrated and prospectively verified.

Large-scale multimodal longitudinal modeling needs to unify the standards of different imaging platforms, molecular detection and environmental measurement. As data dimensionality increases, a sufficiently large sample size is required to avoid overfitting and ensure the universality of the results. In addition, the problems of incomplete data and inconsistent measurement methods will be encountered in practical applications. If some representatives of socio-economic or biological differences are missing from the training data, the deviation of the algorithm may be amplified.

Ethical issues are equally important, such as fair access and data governance. If deployed improperly, those models that rely on a lot of molecular analysis may inadvertently aggravate inequality.

These challenges tell us that in order for AI to play a greater role in OA, we need to work together on methods and clinical research.

## Discussion

Recent AI applications in OA have advanced structural detection accuracy, enabled clinical phenotype prediction, and explored multiomic endotyping and immune-metabolic pathway analysis using machine learning approaches. While these developments have expanded the utility of AI in OA research, they remain largely limited to static cross-sectional analysis or late-stage structural assessment, failing to integrate longitudinal biological and environmental data into a unified predictive framework. Few existing models further incorporate causal reasoning to link early pathogenic dysregulation to actionable preventive interventions, leaving a critical gap in translating AI capabilities to OA early prevention. Our mechanistic forecasting framework addresses this gap by aligning AI modeling with the dynamic, upstream biological processes of OA, repositioning AI from a detection tool to a platform for anticipatory prevention.

Recent research on OA has found that some of our previous basic ideas may need to be reconsidered. Now, through structural imaging technology, we can really see the shape of the disease more clearly. However, seeing more clearly does not mean that we can better predict the development of the disease. Now, scientists are paying more and more attention to immune metabolism disorder and long-term exposure to certain environmental factors, which may be the causes of early disease. If these upstream mechanisms really appear before the obvious structural changes, then the calculation methods that mainly rely on the characteristics of radiographic phenotypes may not be able to help us to intervene in the early stage.

From this point of view, the role of AI in osteoarthritis OA in the future may not be to measure the structural changes more carefully, but to find out the key point-that is, the turning point at which the self-balance ability of organisms begins to be unstable. The important question may no longer be how accurately cartilage wear can be measured, but whether structural damage can be found before it can be seen by X-rays. It is very important to understand the time relationship between this biological imbalance and morphological changes to judge whether AI can really turn OA management to prevention, rather than wait until the late stage to determine the nature.

The biological evolution of osteoarthritis progresses from long-term exposure accumulation to immune and metabolic dysregulation, culminating in irreversible structural damage. Current AI applications are largely confined to radiographic classification of late-stage structural abnormalities. A mechanistic forecasting framework shifts AI deployment earlier in the disease timeline by integrating multimodal biological and exposure data to model dynamic risk trajectories. This repositioning reframes AI from a tool for structural detection to a platform for anticipatory prevention. This figure was created with BioRender (app.biorender.com).
